# Building Sustainable Partnerships to Strengthen Pediatric Capacity at a Government Hospital in Malawi

**DOI:** 10.3389/fpubh.2017.00183

**Published:** 2017-07-27

**Authors:** Michelle Eckerle, Heather L. Crouse, Msandeni Chiume, Ajib Phiri, Peter N. Kazembe, Hanny Friesen, Tisungane Mvalo, Marideth C. Rus, Elizabeth F. Fitzgerald, Allyson McKenney, Irving F. Hoffman, Megan Coe, Beatrice M. Mkandawire, Charles Schubert

**Affiliations:** ^1^Department of Pediatrics, Division of Emergency Medicine, Cincinnati Children’s Hospital Medical Center, Cincinnati, OH, United States; ^2^Department of Pediatrics, Section of Emergency Medicine, Baylor College of Medicine, Houston, TX, United States; ^3^Kamuzu Central Hospital, Lilongwe, Malawi; ^4^College of Medicine, University of Malawi, Lilongwe, Malawi; ^5^Baylor College of Medicine Children’s Foundation Malawi, Lilongwe, Malawi; ^6^Department of Medicine, Division of Infectious Diseases, University of North Carolina at Chapel Hill, Chapel Hill, NC, United States; ^7^Department of Pediatrics, Division of Emergency Medicine, University of North Carolina at Chapel Hill, UNC Hospitals, Chapel Hill, NC, United States; ^8^Department of Pediatrics, Baylor International Pediatrics AIDS Initiative, Baylor College of Medicine-Children’s Foundation Malawi, Lilongwe, Malawi; ^9^Kamuzu Central Hospital/Seed Global Health, Lilongwe, Malawi

**Keywords:** global health, multi-institution partnerships, low resource, resource-constrained, Africa, Malawi, pediatrics

## Abstract

**Introduction:**

To achieve sustained reductions in child mortality in low- and middle-income countries, increased local capacity is necessary. One approach to capacity building is support offered *via* partnerships with institutions in high-income countries. However, lack of cooperation between institutions can create barriers to successful implementation of programs and may inadvertently weaken the health system they are striving to improve. A coordinated approach is necessary.

**Background:**

Three U.S.-based institutions have separately supported various aspects of pediatric care at Kamuzu Central Hospital (KCH), the main government referral hospital in the central region of Malawi, for several years. Within each institution’s experience, common themes were recognized, which required attention in order to sustain improvements in care. Each recognized that support of clinical care is a necessary cornerstone before initiating educational or training efforts. In particular, the support of emergency and acute care is paramount in order to decrease in-hospital mortality. Through the combined efforts of Malawian partners and the US-based institutions, the pediatric mortality rate has decreased from >10 to <4% since 2011, yet critical gaps remain. To achieve further improvements, representatives with expertise in pediatric emergency medicine (PEM) from each US-based institution hypothesized that coordinated efforts would be most effective, decrease duplication, improve communication, and ensure that investments in education and training are aligned with local priorities.

**Call to action:**

Together with local stakeholders, the three US-based partners created a multi-institutional partnership, Pediatric Alliance for Child Health Improvement in Malawi at Kamuzu Central Hospital and Environs (PACHIMAKE). Representatives from each institution gathered in Malawi late 2016 and sought input and support from local partners at all levels to prioritize interventions, which could be collectively undertaken by this consortium. Long- and short-term goals were identified and approved by local partners and will be implemented through a phased approach.

**Conclusion:**

The development of a novel partnership between relevant stakeholders in Malawi and US-based partners with expertise in PEM should help to further decrease pediatric mortality through the coordinated provision of acute care expertise and training as well as investment in the development of educational, research, and clinical efforts in PEM at KCH.

## Introduction

Over the past 15 years, major progress has been made in the fight against HIV, tuberculosis, and malaria in low- and middle-income countries (LMICs), resulting in decreased child mortality rates (1). Despite this, in 2015, 5.9 million children died before their fifth birthday (1, 2). The majority of these deaths were due to either preventable or treatable causes (1–5). Progress must accelerate if the proposed child survival target of the 2030 sustainable development goals (SDGs) is to be reached. The principle requirements for future success in decreasing child mortality will be improvements in local capacity in LMICs and sustained international cooperation through partnerships, both of which depend on training, infrastructure, political will, peace, and the absence of corruption (6).

The World Health Organization (WHO) (7) describes a partnership for health as a means to “bring together a set of actors for the common goal of improving the health of populations based on mutually agreed upon roles and principles.” Multifactorial health issues are ideally addressed through innovative, coordinated, and collaborative initiatives (8, 9) and during the past three decades, an exponential growth of global health partnerships has occurred. Some partnerships are borne of a joint interest, such as engaging in research or accomplishing a specific programmatic goal, while others evolve over time. An ideal partnership is borne from the collaboration of parties working toward a mutual goal. Once successfully established and effectively maintained, these initiatives can be of tremendous mutual benefit, be self-sustaining, and support development of much-needed training programs in resource-limited settings (10, 11).

While many partnerships exist between institutions in high-income countries (HICs) and those in LMIC’s, formal collaborations between US-based institutions are rare and our site was no exception. We describe the evolution of a novel global health partnership from three distinct US-based institutional initiatives. Each program was focused on separate aspects of pediatric capacity building at Kamuzu Central Hospital (KCH), the main government referral hospital for the central region of Malawi, located in the capital city of Lilongwe. It is also the teaching hospital for Kamuzu College of Nursing, Malawi College of Health Sciences, and Malawi College of Medicine (COM). The COM, based in the southern city of Blantyre, has operated a satellite campus at KCH since 2012, which trains third year medical students and clinical officers. The parallel efforts of each institution began to overlap while providing clinical care in this busy inpatient setting with 300–450 pediatric inpatients. With open communication in an environment of mutual respect, our individual initiatives recognized that efforts to build capacity for pediatric care would develop best when working together, and that combining our resources would provide the foundation for collaborative action. We anticipate that long-term resource investment, sustained “on the ground” presence, ensuring that the partnership does not create undue burden on the system, and working cooperatively, with an emphasis on communication among institutions, will result in improved pediatric health systems. We outline the contributions and initial goals of each institution to date, the overlapping aspects of each program, which were collectively recognized, and the plans for moving forward as a multi-institution consortium.

## Background

### Setting

Malawi is a low-income country in sub-Saharan Africa with approximately 17 million people and an average life expectancy of 55 years. It ranks 170 out of 188 countries with respect to its Human Development Index (2016), with an under five mortality rate in 2015 of 63/1,000 live births. HIV prevalence was 10.6% among adults aged 15–49 years (12) and an estimated 130,000 children are HIV-infected. Infections (malaria, respiratory illness, diarrhea, HIV) account for four of the top five causes of death in the country. The predominant local language in the central region, where KCH is located, is Chichewa.

Within KCH, the pediatric ward admits more than 27,000 patients per year and manages all medical emergencies. The department has approximately 215 beds spread over several wards, admitting patients based on acuity, condition, and age. In the rainy (malaria) season, there may be greater than 100 pediatric admissions per day and over 400 inpatients, far more than the ward was built to accommodate. Limitations in various aspects of the system, such as access to supplies and medications, laboratory investigations, and adequate blood bank services provide additional challenges in managing pediatric patients.

In addition to infrastructure, resources, and pharmaceutical supplies, providing adequate care to pediatric patients relies on adequate human resources. In the pediatrics department, there are insufficient specialist pediatricians or generalist doctors to manage the volume of patients, and the burden of clinical care falls largely on mid-level clinical providers. The number of acutely ill patients also overwhelms nursing staff and it is not uncommon for a single nurse to cover a ward of 60 patients or more. In addition, staff often work additional overtime shifts to support the staffing needs of the wards, and these long hours in difficult conditions can lead to exhaustion and burn-out. Malawi has 0.02 doctors and 0.28 nurses per 1,000 population, far below the WHO’s critical level of 2.5 health workers per 1,000 needed to provide adequate care to a population. Various efforts are in place to expand training of additional health workers in Malawi. Improving the staffing shortages at the hospital level will also depend on the ability of the Ministry of Health to employ and retain staff in the public system.

As each of our individual institutions became involved at KCH, it was recognized that the need for clinical capacity building is imperative to allow high-quality educational efforts to take place. All providers observed that the ability to adequately triage or stabilize new admissions was limited, due to both a lack of specific acute care training as well as staffing and resource shortages. Children would enter the ward with varying degrees of acuity and quickly overwhelm staff. Despite improvements, this overall problem persisted; no individual institution had been able to contribute enough resources to consistently change this situation. Therefore, our institutions collectively decided to prioritize pediatric emergency and acute care.

## Institutional Histories

### Baylor College of Medicine (BCM)

The BCM-Abbott Fund Children’s Clinical Center of Excellence (COE) in Lilongwe began providing care to children with HIV in 2005. The COE serves as the outpatient pediatric HIV clinic for KCH and is a pediatric HIV referral center for the country. While the primary focus of the COE is providing care for children with HIV, Baylor has also sought to build clinical capacity for non-HIV infected children, and provided a full-time pediatric hospitalist at KCH from 2006 to 2010. Additionally, BCM COE faculty led an initiative to improve inpatient emergency care utilizing the World Health Organization’s Emergency Triage, Assessment, and Treatment (ETAT) program. This quality improvement effort resulted in decreased mortality in pediatric patients at KCH (13). From the beginning of the COE, Baylor was also involved in managing KCH’s pediatric hematology–oncology program, and currently staffs the inpatient and outpatient pediatric oncology programs at KCH. In 2015, the section of pediatric emergency medicine (PEM) began to send PEM fellows to work clinically at KCH. While primarily working clinically in the pediatric acute care areas alongside staff at KCH, PEM fellows and faculty have also provided bedside teaching for medical students and clinical officer interns from the Malawi COM, as well as participating in hospital initiatives to improve pediatric resuscitations.

### Cincinnati Children’s Hospital Medical Center

The initial relationship between Cincinnati Children’s Hospital Medical Center (CCHMC) and KCH began in 2009 with individual resident trainees undertaking clinical rotations. CCHMC also recognized that there was a need to contribute to the clinical education of Malawian trainees as well as physically supervise our US-based trainees to decrease the strain on clinical operations at KCH and subsequently provided the sustained presence of a supervising pediatrician to KCH in exchange for the ability to continue rotations for US-based trainees. This relationship provided an opportunity for CCHMC, through the division of PEM, to establish specific goals focused on improving medical care and reducing mortality *via* clinical capacity building, enhancing medical education, and initiating collaborative research and quality improvement efforts. There was a specific focus on improved triage and emergency care of critically ill children within the pediatric ward and an improved process for blood transfusions for severely anemic children. In addition, capacity for point-of-care ultrasound was expanded with provision of an ultrasound machine residing on the pediatric ward and supplemental ETAT trainings were sponsored. CCHMC faculty was also integrated into medical student teaching.

### University of North Carolina (UNC)

The UNC has been involved in pediatric research and programmatic endeavors through UNC Project Malawi since 1999, including the initiation of the first Prevention of Mother-To-Child Transmission (PMTCT) program in Malawi in 2001. Research studies have included the evaluation of the efficacy and safety of neonatal HIV prophylaxis, development of algorithms for inpatient pediatric HIV testing and treatment, malaria vaccine trials, and the management of Burkitt lymphoma. UNC physicians have supported pediatric inpatient clinical care at KCH since 2009, and a UNC pediatrician is currently part of the inpatient staff. The UNC pediatric residency program has been sending residents for short-term rotations since 2002 and supported 3 month-long rotations and the fellowship research of a PEM fellow from 2013 to 2016. In 2011, UNC in collaboration with KCH opened a pathology laboratory. This laboratory provides pathology services to all of Kamuzu Central Hospital and the Central Region of Malawi. From 2008 to 2013, UNC partnered with the KCH laboratory through a Centers for Disease Control (CDC) grant, in an effort to improve general laboratory services in the hospital. UNC developed a sickle-cell clinic at KCH in 2013 and has helped establish and maintain Malawian residency programs in Surgery and Obstetrics and Gynecology, and maintains a robust relationship with these departments.

## Simultaneous Initiatives and the Overall Impact of Parallel Efforts

**Figure 1:**
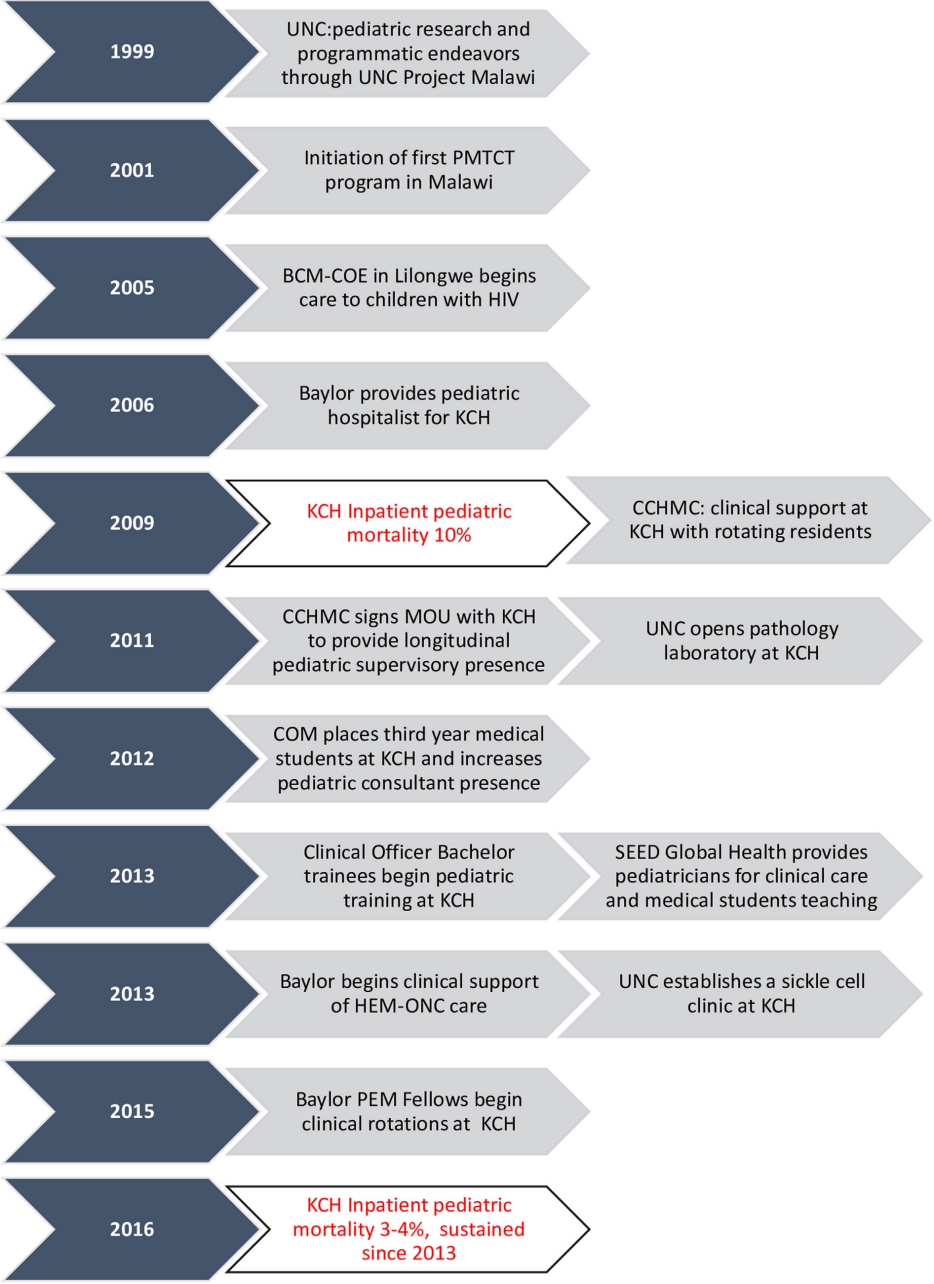
Timeline of institutional activities at Kamuzu Central Hospital.

At the same time that the aforementioned institutions developed individual programs, multiple efforts at improving care were underway from both Malawian institutions and other international organizations. The COM placed third year medical students at KCH for their clinical attachments, including pediatrics. With this effort came increased pediatrician supervision supported by the COM through both the COM itself and other staff assigned from other organizations. To support training of clinical officers (middle level clinicians), another program was instituted to provide specialized pediatric training for experienced clinical officers, with the goal of supporting pediatric care at the district hospital level. Clinical rotations for these trainees were implemented at KCH as well, providing another cadre of trainees who also required bedside and didactic training. Program administration was supported through the COM with additional support from pediatricians allocated through the German Society for International Cooperation (GIZ). In addition, pediatricians placed at COM through the Global Health Service Partnership, a collaboration between Seed Global Health and the US Peace Corps, were instrumental in medical student and clinical officer education as well as clinical care. Figure 1 shows the timeline of the various institutional contributions at KCH.

These programs and the support of outside institutions significantly improved the clinical care within the pediatric ward, supporting the fact that expansion of training programs offers benefits, which extend beyond the trainees themselves. Clinicians from each of the above programs worked alongside each other to develop specific initiatives, such as restructuring the space allocated for acute care to improve flow, instituting use of a tracking system for children needing blood transfusions, and providing the ancillary staff with training in the early identification of critically ill children. The combined efforts of local staff and foreign partners led to a dramatic decrease in inpatient mortality, from >10% in 2009 to the current rate of <4% in 2016.

## Collaboration as a Way Forward: Pediatric Alliance for Child Health Improvement in Malawi at Kamuzu Central Hospital and Environs (PACHIMAKE)

Despite these improvements, there was friction at times between the different stakeholders. Lack of coordination led to duplicative efforts. At this point, US stakeholders recognized that this lack of communication and coordination was affecting clinical care and outcomes and at times frustrating local providers. With the high acuity of illness and the high inpatient mortality, it was decided that a more collaborative approach would be required to further improve outcomes. Therefore, a cooperative effort was embarked upon which elevated the care and survival of children above the priorities of individual institutions and demanded transparency and cooperative policies. In 2015, a working group was formed to begin discussions and outline future goals aimed at improving pediatric care at KCH. This group chose the name PACHIMAKE, a Chichewa word meaning “the heart of the matter.”

Once the working group from the three US institutions was formed, a site assessment was planned and then completed in November 2016. US and local stakeholders met multiple times to identify critical gaps that remained in pediatric care at KCH. Issues identified by local staff were given highest priority when determining the next steps for the consortium. Priority items identified for further work included reliable systematic data collection, adequate staff (specifically nurses and middle-level clinicians), coordination among institutions sponsoring rotating consultants and trainees, standardization of clinical care, and appropriate core infrastructure (Table 1). Of note, these priorities came directly from our Malawian partners. Solutions were proposed and short- and long-term goals were developed to collaboratively close the identified gaps in care. The past collaborative work between the US and local Malawian institutions had prepared us to begin work on mutually identified goals in a phased approach in the upcoming years.

**Table 1:** Key consortium practices and goals.

Pediatric Alliance for Child Health Improvement in Malawi at Kamuzu Central Hospital and Environs Core PracticesImproving child survival at KCH is the overall goal, which all consortium actions serve, guided by principles of: Transparency Open respectful communication Shared code of conduct Mutual professionalism Joint recognition of successes/shared authorship with Malawian colleagues	

**Areas of focus identified by local partners**	**Proposed solutions**

Reliable data system	Support implementation of electronic medical record, identify priority metrics for assessment of care based on local published standards (Council for Health Service Accreditation of Southern Africa)
Adequate staffing	Support staffing needs by leveraging resources from US-based institutions, provide consistent consultant presence year-round to support supervision and education of Malawian trainees
Coordination of US clinical rotators	Maintain open communication between institutions, guided by local needs and assuring outside rotators complement rather than burden the system
Standardization of clinical care	Support staff training in emergency care, develop and implement KCH-specific protocols for common conditions
Improvement of core infrastructure	Continue seeking funding opportunities to support acquisition and maintenance of equipment and medications and facilities

We believe that directing efforts toward locally identified goals is one of the strongest aspects of this partnership. Because each institution is deeply committed to clinical care at KCH, we recognize that these priorities must be accomplished in order to realize the goals of the PACHIMAKE partnership. This is also in alignment with best practices highlighted by existing global health partnerships supported by the US government (14). This required honest, transparent discussion, and logistical consideration. Given the time and effort invested by each individual institution, there were naturally some perceived threats to each one’s investment as we moved toward collaboration. Table 2 describes pitfalls encountered as the consortium has formed and the lessons learned and strategies used. We have prioritized transparency among all partners. We have developed a code of conduct to support professionalism and ensure the continued collegiality of the group. We maintain a database of ongoing research efforts and potential projects to foster open communication and collaboration. We have discussed authorship on future projects, with the intent to have a “working group” for all efforts and support the development of Malawian clinicians as primary investigators in consortium research. In addition, we have set as our benchmark the improvement of survival and health of the children at KCH to guide all of our collaborative efforts.

**Table 2:** Pitfalls and lessons learned/strategies for partnership development.

Pitfall	Lessons learned/strategies
**Past and Present US Institutional Agendas** Each institution entered with a different history with the host site and is beholden to the priorities of US-based institutional leadership. US-based institutions had previously initiated projects without local input	Use PACHIMAKE formation to set new priorities guided by the host site and present this consortium and goals back to US-based leadership to support investment Leverage existing infrastructure in-country to expand scope of clinical services provided
**Past history between institutions (US-based and local)** Previous interactions between institutions, both historical and experienced by PACHIMAKE members, led to some wariness about the extent of collaboration	Develop memorandum of agreement Implement a formal code of conduct to guide inter-institutional endeavors Assemble a “working group” credited and named for any scholarly work Focus on shared goal of reducing child mortality
**Burden on system created by US-based institutions** Short-term faculty rotators and variation in trainee preparedness created a burden for the host institution	Provide a long term US faculty at the host site Commit to longer rotations at the host site for US-based faculty/trainees Standardize orientation for US-based rotators Coordinate consultant/faculty presence with local learners for increased shoulder-to-shoulder mentoring
**Communication challenges** Challenges included both logistical issues as well as issues of transparency and cross-cultural barriers	Lead with and maintain transparency in all activities Implement monthly calls/meetings including all partners Ongoing assessment of partnership benefits and burdens—honest communication about contribution of partnership to host site

While this partnership is in the early phase, we present our account of its formation as an example for US-based institutions who find themselves working alongside others at a common host site. We encourage a conversation of how individual goals intersect and whether those goals would be better achieved through collaboration. The global health funding climate is now more supportive of multi-institution partnerships; if this collaboration is successful, we anticipate scaling up collaboration among different sub-specialty service lines within pediatrics. As we progress, our outcome indicators of success will include ability to accomplish the goals set forth by KCH staff, coordinate support for a pediatric residency training program, provide continuous specialist (PEM) coverage at KCH, and document progress through presentations and publications.

Our vision is that the coordinated efforts of this multi-institutional consortium will significantly contribute to our overall goal, decreased morbidity, and mortality among children at KCH. Each partner has secured administrative support for this consortium from their respective institution and next steps now include identifying funding sources for the highest priority initiatives. We plan to prioritize reliable data collection and anticipate that this will direct the consortium’s efforts in terms of prioritizing projects for initial funding, which will be pursued at both the individual institutional level and collaboratively through both foundations and government. We anticipate that this consortium could serve as a model of collaboration for HIC institutions striving to improve pediatric health-care systems in other LMICs.

## Conclusion

In the absence of any other identifiable internal changes or external conditions, the improvements in care and decrease of mortality in pediatric patients at KCH can be attributed in large part to the clinical changes brought about by the training and educational initiatives resulting from the partnerships of the above US-based institutions with KCH, the Ministry of Health (MOH), and the COM. In order to sustain and improve upon these gains, investments focusing on advanced care of emergently ill children are needed, as the majority of childhood mortality still occurs in the first 48 h of hospital admission. The development of a partnership between relevant stakeholders in Lilongwe (KCH, MOH, and COM) and US-based partners with expertise in PEM (CCHMC, BCM, UNC) should help to further decrease mortality through the provision of acute care expertise and training as well as investment in the development of educational, research, and clinical efforts in PEM at KCH. In order to continue on the path of fulfilling the millenium development goals and to eventually reach the SDGs collaboration for the good of children and health for all people in general must become the norm and not the exception. Our demonstration of a collaboration that puts the goal of improved health and survival of children over individual institutional goals can stand as a model for others. The opportunities in child health to make the whole of our efforts when working together greater than the sum of our individual efforts are myriad. We must only align our priorities together. The health of the world’s children depends on it.

## Author Contributions

All authors listed provided substantial contributions to the development of the partnership described in this manuscript, provided critical feedback on manuscript drafts, approved the final version of the manuscript, and agreed to be responsible for content.

## Conflict of Interest Statement

The authors declare that the research was conducted in the absence of any commercial or financial relationships that could be construed as a potential conflict of interest.

## Abbreviations

LMICs, low- and middle-income countries; SDGs, sustainable development goals; WHO, world health organization; HICs, high-income countries; KCH, Kamuzu Central Hospital; COM, college of medicine; BCM, Baylor College of Medicine; COE, Baylor College of Medicine -Abbot Fund Children’s Clinical Center of Excellence; ETAT, Emergency Triage Assessment and Treatment; PEM, Pediatric Emergency Medicine; CCHMC, Cincinnati Children’s Hospital Medical Center; UNC, University of North Carolina; PMTCT, prevention of mother-to-child transmission; CDC, centers for disease control; GIZ, German Society for International Cooperation; PACHIMAKE, Pediatric Alliance for Child Health Improvement in Malawi at Kamuzu Central Hospital and Environs; MOH, Ministry of Health; MDGs, millenium development goals.
